# Retrospect of Materia Medica, and Therapeutics

**Published:** 1846-10

**Authors:** J. M. Neligan

**Affiliations:** Lecturer of Materia Medica in the Peter street School of Medicine; one of the Physicians to Jervis street Hospital


### RECORD OF MEDICAL SCIENCE.

*Retrospect of Materia Medica, and Therapeutics.* By J. M. Neligan, M. D., M. R. I. A., Lecturer of Materia Medica in the Peter street School of Medicine; one of the Physicians to Jervis street Hospital.

*Alkalies as a Remedy in Cutaneous Diseases.*—M. Devergie has recently published some interesting observations on the alkaline treatment of skin diseases. He has employed alkalies in both papular and scaly affections; but with most success in the former, and particularly in the various forms of lichen. He employs three salts: the bicarbonate of soda, the carbonate of soda, and the carbonate of potash. The first of these he administers only internally, and usually prescribes it in solution, in some mild stimulant bitter infusion, or in carbonic acid water, the latter being an imitation of Vichy water. The dose at first is fifteen grains daily, in three or four glasses of the infusion, and this dose is augmented by eight grains every third day, until it arrives at one drachm, which dose is not exceeded. Externally the alkaline treatment is used in four different forms: in baths, in lotions, in powder, and in ointment. For the preparation of baths, either the carbonate of soda or carbonate of potash is employed—the quantity used for a single bath varying from eight to sixteen ounces, the strength being gradually increased. For scrofulous or debilitated individuals, he recommends the addition of one pound of common salt to each bath. The alkaline lotions are found of special benefit in skin diseases affecting parts covered with hair, as in the scalp, where they are usually so obstinate. For a lotion, from two to three drachms of carbonate of soda are dissolved in a pint of water. To the benefits derivable from the use of this alkaline wash in chronic eczema and impetigo of the scalp, we can bear testimony, from an extensive experience of its employment, both in hospital and in private practice. The alkalies are used in the form of powder, as a depilatory, in tinea and in sycosis mentis. M. Devergie, however, employs the alkalies chiefly in the form of ointment, and sometimes combines a little quicklime, or a little sulphur, with them. He uses ointments of different strength, according to the nature of the disease. Thus, for lichen and its forms, the proportion is from eight to fifteen grains of carbonate of soda to the ounce of lard; for lepra, psoriasis, or icthyosis, fifteen to thirty grains to the ounce of lard; and for porrigo favosa, thirty to sixty grains, with a grain or two of quicklime. It must be remembered, that the carbonate of potash is more caustic than the carbonate of soda. The following are some of the formulæ he employs:—*Alkaline Liniment*,—Carbonate of soda, ℥i.; olive oil, ℥iv.; the yolk of one egg; first moisten the carbonate of soda, and then incorporate it with the oil and yolk. *Alkaline Syrup.* —Bicarbonate of soda, ℥ss.; simple syrup, ℥viii.; dose, a teaspoonful, morning and evening, in a glass of water. *Alkaline Powder.*— Carbonate of soda, in an impalpable powder, one part; fine starch, ten parts. For external use only.

*Ammonia as a Vesicant.*—The strongest solution of ammonia has been much employed on the Continent, particularly in France, for some years back, as a speedy blistering agent, but has been very little used hitherto in this country. Various directions are given, as to the mode in which it ought to be employed, but Dr. Gondret's ointment is the preparation in most general use, and is the one most highly spoken of. As prepared according to the formula originally published by him, it has been found in many instances not to act satisfactorily. The inventor of it, in consequence, recently made public the following formula for its preparation:—Take of axunge, ℥i.; oil of sweet almonds, ℥ss.; melt together with a gentle heat; pour the mixture, while still liquid, into a wide-mouthed glass vessel; then add, solution of caustic ammonia, ℥v., and mix with constant agitation till cold. Particular care must be taken that the axunge be merely melted; if it be too fluid, or too warm, some of the ammonia will be vaporized, and the resulting ointment too weak. The ammoniacal ointment, thus prepared, retains its properties for many months, if kept in stoppered glass bottles in a cool place. Gondret's blistering ointmeut produces vesication in about ten minutes; it is applied, by spreading it on the skin, and covering the part with a compress. The French use it most frequently for blistering the temples in diseases of the eyes. The rapidity and certainty of its action, however, renders this vesicant of great value in many diseases which need not be enumerated here.

*Ammonia as a Remedy in Asthma.*—M. Rayer has recently published his experience of the effect of strong water of ammonia applied to the velum palati for the cure of asthma. M. Monneret and others had previously employed this mode of treatment, but they applied the caustic to the back part of the pharynx, and in some instances death had nearly ensued from suffocation, owing to the action of the volatile alkali on the glottis. M. Rayer's method of employing this remedy is as follows: he dips a roll of lint, about the length of the middle finger, in a mixture of four parts of strong aqua ammoniæ and one of water, pressing out the superfluous liquid, and immediately applies it for a few seconds to the velum palati, as if about to cauterize the part. The patient is immediately seized with a feeling of suffocation; a fit of coughing ensues, with much expectoration, and this is soon followed by a great feeling of comfort and facility of respiration. Should any return of the fit occur on the day following, the ammonia is again applied. The degree of tolerance of this remedy by patients varies very much; it is, therefore, always well to use it weak at first, which is easily done by moving the piece of lint, dipped in the solution, three or four times rapidly through the air, and then smelling it, when the strength is readily ascertained. In M. Rayer's experience, extending to over a hundred cases, a single application rarely failed to afford relief, and in many instances prevented a return of the attack for three or four months. This mode of treatment is alone applicable to simple or idiopathic asthma, that form which is so often dependent on emphysema, and is attended with catarrh; it has, nevertheless, afforded relief in some cases of symptomatic asthma.

*Arseniate of Quina.*—This salt, first prepared by M. Bourières, has latterly been much used in France in the treatment of obstinate intermittents, and, it is stated, with much success; the chief obstacle to its more general employment being, according to Dr. Boudin, its extreme bitterness. It is readily prepared as follows: Dissolve half an ounce of sulphate of quina in boiling water, and precipitate it with ammonia; wash and dry the precipitate, and dissolve it with the aid of heat in three ounces of distilled water, containing two scruples of arsenious acid in solution; as the solution cools, crystals of arseniate of quina are deposited, which are to be dissolved in distilled water and recrystallized. It is a light, white salt, crystallized in brilliant satinny needles. It is soluble in water, but more so in boiling than in cold water; it is also soluble in weak alcohol, but is insoluble in absolute alcohol or in ether. The dose of it is from one to two grains in divided doses in the course of twenty-four hours. It is usually given in solution in distilled water, to which a little simple syrup may be added.

*Belladonna.*—An ointment consisting of one part of the extract of belladonna to three of lard, has been used with much benefit by Dr. Phillippe, chief surgeon to the Military Hospital at Bordeaux, for the cure of inflammation of the testicle, whether arising from direct injury or as the result of urethritis. He employs it in every stage of the disease, but states that he finds it most useful when the acute inflammatory symptoms have been previously subdued by antiphlogistic treatment, or in cases where induration and thickening of the epididymis remain after other treatment. About half a drachm of the ointment, prepared as above described, is rubbed into the scrotum twice daily, the inunction being continued for five minutes each time. The mean period of cure was five days in thirty cases thus treated. Dr. Phillippe also employs this ointment with most beneficial results in the treatment of buboes.

In the incontinence of urine in children, Dr. Morand has administered extract of belladonna internally, with almost invariable success. He prescribes it in the form of pill, each pill containing a fifth of a grain of the extract. For children from four to six years old he orders one of these pills to be taken night and morning; if at the end of eight days no effect is produced, he directs a third to be taken in the middle of the day. If, after fifteen days, there is no improvement, a fourth pill is added at bed-time, but the poisonous effects of the drug must now be closely watched, as they are often suddenly developed. For children of the age of eight, twelve, or fifteen years, three pills daily are at first administered, and the quantity, at the end of eight days, gradually increased to six. Even this latter number is sometimes exceeded; eight, ten, twelve, or fifteen pills daily being often requisite to effect a cure in youths. In Dr. Morand's practice, from two to four months' use of this remedy are ordinarily sufficient to produce a radical cure of this intractable malady.

Schroeder has employed the vapour obtained by burning belladonna leaves to check hemoptysis. From a drachm to a drachm and a half of the dried and cut leaves is thrown on red hot coals, and the patient respires the fumes as they arise. This simple remedy has, in Dr. Schroeder's hands, seldom failed to check hemorrhage from the lungs.

*Benzoic Acid.*—A few years since Dr. Ure, of London, reasoning on Liebig's theory of the conversion of uric into hippuric acid by the action of benzoic acid when taken into the stomach, recommended the latter as a remedy for calculous diseases where uric acid predominates, and this proposal led to its adoption in practice to a certain extent. Soon after, however, Keller published, in Liebig's Annals of Chemistry and Pharmacy, the result of some experiments on himself, by which it was proved that the supposition of Dr. Ure was false, inasmuch as the urine of individuals who had taken benzoic acid, still contained the usual amount of uric acid after the separation of hippuric acid. More recently, Messrs. Booth and Boyé have investigated this subject anew in America, and the results of their experiments may be stated thus:—1st, The formation of uric acid in the healthy urine is not affected, either in regard to its quantity, or its external properties in general, by the introduction and transformation of benzoic acid into hippuric acid in the system. 2d, The time required for the benzoic acid to pass through the system, and reappear as hippuric acid in the urine, is from twenty to forty minutes after its introduction with food into the stomach; its occurrence continues for four or eight hours, but then ceases. 3d, The quantity of hippuric acid obtained from the urine is greater than that of the benzoic acid taken. In round numbers it may stated to be one-third more. 4th, Urea is not in combination with hippuric acid in the urine. From these results it is evident that benzoic acid is not to be looked upon as a remedy for uric acid diseases.

*Bromine and its Preparations.*—The very high price which iodine has attained within the last twelve months, has rendered it very desirable that a substitute should, if possible, be obtained for this medicine, which is at present so extensively employed. Bromine and its preparations have been shown by the experiments of Magendie, Barthez, Brame, and others, to possess therapeutical properties as nearly as possible identical with those of iodine and the iodides. The scarcity, however, of bromine, and consequently, its commercial value, has hitherto prevented its general employment as a remedial agent; but the recent discovery of it in large quantities in America has recalled attention to this substance as a substitute for iodine. Mr. O'Reilly, of this city, while lately in the neighbourhood of New York, having had his attention called to the peculiar properties of the mother waters of many brine springs in the United States—the result of their evaporation for procuring common salt—found by experiment that they contained bromine in large quantities, nine drachms in every gallon. Having procured a large amount of bromine from this source, he has brought a hundred pounds weight of it home, and states that he can obtain an almost unlimited supply of it: the price at which it is now sold in Dublin is eighteen pence an ounce. These circumstances have induced us to include in our retrospect a short account of the doses and mode of administration of bromine, and its preparations.

The forms in which it has been used on the Continent are, in the simple state much diluted, and combined in the form of bromides with potassium, barium, calcium, iron, and mercury. These preparations are made by processes exactly similar to those used for procuring the corresponding combinations of iodine. As a substitute for the tincture of iodine, M. Pourche has employed the following solution: bromine, one part; distilled water, forty parts; dose, from five to six drops in some aqueous vehicle, three or four times daily. For external use he employs a solution four times as strong as this. The *bromide of potassium* is very soluble in water, sparingly soluble in alcohol; the dose of it is from four to eight grains three times a day: to prepare an ointment from it, four parts are rubbed up with thirty-two parts of lard; and if a stronger ointment, or one resembling the compound iodine ointment, be wished for, six drops of bromine are added to this. The *bromide of barium* is also soluble in water; the dose of it is from one to five grains three times a day: the ointment is prepared by combining it in the proportion of one part to ten of lard. The *bromide of calcium* is prescribed in the form of pill made with the conserve of roses; the dose of it is from three to ten grains. The *bromide of iron* is a brick-red deliquescent salt, very soluble in water; it is not so easily decomposed as the iodide of iron, and is given usually in the form of pill made with conserve of roses and gum arabic; the dose of it is from one to three grains: it has been employed externally also in the form of ointment, prepared with one part of the bromide to fifteen of lard. Two *bromides of mercury* have been used: the first, a sub-bromide, is a white insoluble powder; the dose of it is one to two grains daily: the second, a bromide, is fusible and volatile, and soluble both in water and alcohol; its dose is one-sixteenth of a grain, gradually increased to one-fourth of a grain, daily. All the preparations of bromine may be readily known from those of iodine by their not disengaging violet-coloured vapours when concentrated sulphuric acid is poured on them.

In France, bromide of potassium has been of late fraudulently sold for iodide of potassium, in consequence of the high price of the latter; a sophistication of but little importance, if, as we are inclined to believe, the medicinal action of both be identical.

*Camphor.*—An adulteration of this substance with muriate of ammonia has been lately detected in Brussels, and is said not to be uncommon in France; we are not aware that the fraud has been practised in British commerce as yet. It may be readily detected by the action of quick-lime, which would liberate the ammonia; or by treating a suspected specimen with water, which would dissolve out the muriate of ammonia.

At a late meeting of the *Societé Medico-pratique* at Paris, many of the members cited facts tending to prove that camphor is a medicine the abuse of which is extremely dangerous. M. Homolle related a case of phthisis in which he prescribed more than twenty grains of camphor, in divided doses, in the twenty-four hours; the effect of which was, that the patient was attacked with frightful dyspnœa, continued nausea, and violent palpitation of the heart, all of which symptoms were with much difficulty subdued. Dr. Gaide mentioned the case of a man who was in the habit of taking camphor in very large doses, as a consequence of which he became affected with aggravated diphtheritis. M. Moreau stated, that he had seen a lady attacked with acute meningitis, which only yielded to the most active treatment, from having taken large doses of camphor to cure an obstinate neuralgic affection. Dr. Labarraque said, that a butcher, for whom he had prescribed six grains of camphor, was attacked with violent vomitings which nearly proved fatal.

*Castor Oil.*—The mildness and certainty of operation of this cathartic give it peculiar advantages in the treatment of many diseases; very often, however, its tendency to produce vomiting prevents it from being employed. To remedy this inconvenience, M. Parola proposes the substitution of an extract, an ethereal, and an alcoholic tincture of castor-oil seeds, for the oil itself. The result of his experiments on himself and on numerous sick and convalescent individuals is a follows:—1st. That the ethereal and alcoholic tinctures have a purgative action four times as strong as the oil obtained by expression, and that they are not so apt to produce vomiting, nor so irritant as the ordinary oil. 2d. That these new preparations remain unalterable for a long period without reference to climate or season. 3d. That the ethereo-alcoholic extract possesses a purgative action comparatively weaker than the marc or pulp from which it is extracted, proving that the seeds contain a principle which is insoluble in alcohol or ether. 4th. The advantage of the new preparations, so far as relates to their not causing vomiting, is easily explained by the smallness of the dose in which they are administered.

M. Righini has directed much consideration to the divising of a formula for prescribing castor-oil, and the following form, in which the purgative properties are not in the least diminished, he states to be free from the usual inconveniences of a dose of this medicine :—Take of finely-powdered gum-arabic, ʒii. ; pure water, ℥iii. ; make a mucilage with a small quantity of the water, and then add of castor-oil ℥i. ; mix carefully, and afterwards pour in, while agitating the mixture, the rest of the water ; finally add, with constant agitation, the filtered juice of one orange, and one ounce of simple syrup.

*Carragheen Moss.*—Dr. Frank, of Wolfenbuettel, employs a compound powder of Irish moss as an article of diet for phthisical patients, and for children affected with tabes mesenterica. It is prepared as follows, and has a most agreeable taste :—Take of Carragheen moss, cleaned, ℥ss. ; spring water, ℥xvi. ; boil down to one-half ; strain with expression ; and add to the strained liquor, white sugar, ℥iv. ; gum arabic, in powder, ℥i. ; and powdered orris-root, ʒss. ; heat to dryness with a gentle temperature, stirring constantly, so as to obtain a pulverulent mass, to which three ounces of arrow-root are to be added with trituration. A jelly is prepared with this powder, by rubbing up a tea-spoonful of it with a little cold water, and then pouring a cupful of boiling water on it.

*Iron.*—The combinations of this metal with the vegetable acids have been much employed in medicine of late years, and many practitioners prefer them to the older preparations—the sulphate and muriate. Bouchardat has recently laid down the two following propositions with reference to the forms in which iron should be prescribed. 1st. That it should be either in the state of protoxide or in that of the pure metal, which is converted in the stomach into a salt of the protoxide ; and 2d, that the protoxide should be united to carbonic, or to some other organic acid which is capable of being assimilated. In compliance with these propositions, the best preparations of iron are, amongst the insoluble, iron reduced by hydrogen and the carbonate of the protoxide ; and amongst the soluble compounds, the lactate and citrate of the protoxide. The three latter are at present very generally prescribed in this country, and consequently ordinarily to be met with in apothecaries' shops ; but the use of the former is as yet confined to the Continent, where it is held in high esteem. The employment in medicine of iron reduced to the state of minute division, by means of hydrogen, is due to the observations of MM. Quevenne and Miquelard. To obtain it, a certain quantity of black oxide of iron (*Æthiops martis*) is introduced into a tube of porcelain, which is heated to redness ; and a current of hydrogen gas is then passed over it until it is reduced, which ordinarily occurs in from seven to eight hours. The chief circumstance to be attended to, during the operation, is the state of the temperature. If it be not sufficiently high, the reduction does not take place ; and if it be too high, the iron is reduced, but is agglutinated into ductile plates. For preparing it on the large scale, a metal water-pipe is employed, and the oxide is placed on numerous small shelves made of sheet iron and supported on small iron bars. When properly prepared, *reduced iron* (*fer rêduit*) is in the form of a fine light powder, of a bright greyish slate-colour, in very minute division, and free from any trace of sulphur. The advantages which iron in this state possesses as a therapeutic agent are, first, that it is readily acted on by the weak acids—the lactic and muriatic, which are ordinarily present in the gastric juice during digestion; and second. that it is free from the inky taste which the preparations of iron possess in a degree proportioned to their solubility. The dose of it is from one to ten grains; it may be given in the form of pill or of bolus. The French physicians usually prescribe it made into pastilles with chocolate.

*Iron Filings.*—It has been always found a matter of much difficulty to preserve iron filings without their becoming oxidated. M. Giovanni Righini has discovered that they may be preserved for an indefinite period, even in paper, by first triturating them with an equal quantity of very dry sugar.

*Iodide of Iron.*—M. Cop has proposed the following very simple process for preparing the iodide of iron. Bruise together in a large mortar four parts of iodine and two parts of water; then add quickly one part of iron filings. Sufficient heat is produced to drive off one part of the iodide in the state of vapour; the mixture becomes liquid; to remove the excess of iron it is to be dissolved in water and filtered. The filtered liquid is a solution of the iodide of iron, free from oxide or peroxide. This solution may, of course, be readily preserved by adding a sufficiency of pure sugar to it to convert it into a syrup.

*Mercury.*—From the result of numerous experiments, M. Bouchardat draws the following conclusions with reference to the activity of the salts of mercury. Of the soluble compounds, the most active is the red iodide, rendered soluble by means of iodide of potassium; next to it, corrosive sublimate; and then the cyanuret. The activity of the insoluble compounds is in the following order: the red iodide, precipitated calomel, the yellow iodide, sublimed calomel, and metallic mercury. Bouchardat's experiments have been principally made on fishes; but his results agree very closely with the opinions of most therapeutists, and particularly with those of M. Trousseau.

*Myrrh.*—No analysis of myrrh having been published since that of Braconnot, which, from the small quantity of resin indicated by him, was manifestly imperfect, Ruickoldt has reinvestigated the chemical history of this substance. The myrrh which he analysed consisted of irregular, knotty, roundish, tear shaped pieces, of the size of a hazel nut; its color was yellowish, with a reddish or even darker tinge. Its fresh fracture had a waxy lustre, in some places resinous, with white, opaque striæ, and amygdaloid indentations of the same colour. Its specific gravity was 1.120 to 1.180. One hundred parts were composed of :

| | |
|---|---|
| Volatile oil (*myrrhol*) - - - - - - - | 2.183 |
| Resin (*myrrhin*) - - - - - - - | 44.760 |
| Gum (*arabin*) - - - - - - - | 40.818 |
| Water - - - - - - - - - - | 1.475 |
| Impurities - - - - - - - - - | 3.862 |
| Carbonates of lime and magnesia - - - - - | 3.650 |
| Gypsum and oxide of iron - - - - - - - | a trace. |
| | 96.748 |

Myrrhol undergoes decomposition on exposure to the air merely : it is thick, of a wine-red colour, and of a penetrating odour ; is lighter than water, and is readily dissolved by ether and by alcohol. Its composition is $C^{44}H^{33}O^{4}$ differing scarcely from colophony and from sylvic acid. The resin is completely soluble in ether, but imperfectly so in alcohol; its solution has no action on turmeric paper; when heated to 336° F. it furnishes a very acid transparent liquid which has been named by M. Ruickoldt *myrrhic acid*. The composition of *myrrhin* $C^{48}H^{32}O^{10}$.

*Nitrate of Silver in Hooping-Cough.*—The following mode of treating hooping-cough has been very successful in the hands of M. Berger. In the first stage he employs moderate antiphlogistic treatment, purgatives and repeated emetics, particularly ipecacuanha in combination with tartar emetic. In the convulsive stage, in which the indication is to combat nervous irritation, not being satisfied with the results that he obtained from the use of the remedies ordinarily employed, he was induced to administer nitrate of silver, the effects of which, he states, are singularly beneficial. He prescribes it in doses of from a sixteenth to a twelfth of a grain three times daily at first, and afterwards four times a day ; of course, it should not be given in cases where the state of the digestive organs contra-indicates its employment.

*Oils.*—M. Mahier has recently published some interesting observations on the action of bitter almonds, cherry-laurel leaves, peach blossoms, and their distilled waters, on the aromatic properties of essential oils. The observations first made on the action of the syrup of almonds in destroying the odour of musk, and since confirmed by M. Soubeiran, and, latterly, the effects of cherry-laurel water in similar circumstances, discovered by M. Fauré, of Bordeaux, induced M. Mahier to undertake the generalization of this reaction on essential oils, and on other strongly odorous substances. "Although," he says, "the result may not contribute much to scientific knowledge, they, nevertheless, possess some practical interest, if it be only in affording a quick and easy method of purifying bottles and other vessels from odours which it is found difficult to remove by other means. Having recently tried to remove, by means of vinegar and then of ashes, the smell from a marble mortar which had been used in the preparation of an enema of assafœtida, the result being imperfect and unsatisfactory, I tried the use of bitter almond paste, the residue of some that had been used for preparing the syrup. Having rubbed a little of this in the mortar without its having the effect of removing the odour, I added a little water, so as to develope the bitter-almond odour; I rubbed it again, and then washed out the mortar with a good deal of water, by which means the odour of the assafœtida was completely removed. This first trial induced me to apply this method to the cleansing of vials and bottles which had contained spirits of camphor, oil of spike, essences of cloves, mint, neroli, lavender, citron, and turpentine, oils of petroleum, copaiba, cod-liver, creasote, and different odorous balsams, and resinous tinctures. All the bottles were rendered completely clean, void of odour, and as good as new. It is necessary, in cases where the vessels previously contained fatty matters, to cleanse them with cinders or potash, and to rinse out with spirit those which had contained resinous or balsamic tinctures, before using the almond paste. A few cherry-laurel leaves, or peach-blossoms, beaten into a pulp, and introduced into the bottles, produce the same effect as the bitter almonds. The same de-odourising action, it is fair to suppose, is possessed by all leaves and flowers containing prussic acid, and probably also by other strong-smelling substances.

*Pomegranate-root Bark.*—This substance, highly praised in the East and on the Continent of Europe, as a vermifuge in cases of tape-worm, enjoys in this country but a limited reputation. That the cause of this bad repute of the remedy is altogether owing to the mode in which it is prescribed we have been long convinced; we therefore lay before our readers the recently published observations of Dr. Mérat, who has been in the habit of using the root-bark of the pomegranate in his practice for the last twenty-four years, in which time, he states, he has never found it fail in curing tape-worm. To ensure success, he affirms that attention to the following conditions is indispensable: first, that the medicine should not be administered except on the day, or the day after that in which joints of the worm have been passed; second, that the individual should take in three doses, with an interval of half an hour between each dose, a decoction of two ounces of the *fresh* root of the cultivated pomegranate in twenty-four ounces of water, boiled down to nineteen ounces.

*Savin.*—As an application to venereal vegetations, Vidal (de Cassis) recommends a combination of one part of powdered savin, and two parts of finely-powdered alum. It is sprinkled over the vegetations, and the prepuce then drawn forwards; but where this is not possible, simple dressing is applied. The application is renewed twice daily.

*Senna.*—An interesting account of the natural history of this valuable medical plant has been recently published by M. Landerer of Athens. It is chiefly indigenous in Ethiopia, Arabia Felix, Abyssinia, Nubia, and Sennaar. The Arab tribes who occupy themselves with this branch of commerce, do not pay the least attention to the cultivation or management of the plants. The senna plant attains the height of eight or ten feet, and affords to the inhabitants of the Desert some protection from the heat of the sun. The senna harvest begins about the end of September. The Arabs cut nearly all the branches off the trees, and expose them to the sun until the leaves begin to fade, when they are placed on high ground, and on rocks, so as to be dried as quickly as possible. As soon as they are dry, the branches are laid in heaps and beaten with sticks to shake the leaves off. The leaves obtained by this process are not damaged, and consequently fetch the highest price, nearly double the sum given in the bazaars for the broken senna. As all the leaves are not separated by this means, the branches are, in some parts of Nubia, placed on a dry floor, and camels driven over them; the remainder of the leaves are thus obtained, but they are much broken, and small pieces of the stems are mixed with them. The senna collected in various parts of Africa is packed in linen sacks, and conveyed on camels in caravans to the shores of the Nile, where it is transferred to boats, and brought thus to Cairo and Alexandria. In both these capitals there are senna magazines, to which the bales are conveyed to be unpacked, and again carefully sorted. Within the last two years the senna trade was thrown open, but it has latterly again become a government monopoly. An intentional adulteration of senna with other leaves is, in their native country, out of the question, for the slightest adulteration is there punished as a capital crime. The fruit, which is rarely found mixed with the leaves, because it is carefully picked out, is in very general use in the countries where senna grows. Two varieties of senna are ordinarily met with in the bazaars of Constantinople and Smyrna; an Egyptian and a Tripolitan product.

*Turpentine.*—The following physiological effects of oil of turpentine have been noticed by M. Bouchardat while lately engaged in some experiments on this substance, during which he was exposed for five or six hours at a time to the inhalation of the atmosphere of the laboratory charged with its vapour. The effects were in no instance manifested until night, at the usual hour of repose. They consisted in sleeplessness, constant restlessness, heat of skin, the beats of the pulse increased from sixty-five to eighty-six in the minute; some difficulty in passing water, which possessed in a remarkable degree the characteristic turpentine odour, and on the following day very great lassitude, accompanied by pain and a feeling of weight in the region of the kidneys. The lassitude, debility, and inability to work, continued for two or three days afterwards. M. Bouchardat is of opinion that it is in consequence of habit removing their susceptibility, that painters, furniture-varnishers, and others exposed to the vapour of turpentine, do not suffer from these effects of its inhalation.

*Valerianic Acid and the Valerianates.*—Prince Louis Buonaparte was the first to call the attention of physicians to this acid and its preparations; but the process proposed by him for its preparation having been found expensive, and not applicable to the procuring of it on the large scale for use in medicine, a number of methods for preparing it have been published, both by Italian and French chemists. The Pharmaceutical Society of Paris recently requested a report on the different processes from MM. Cap, Louradour, and Blondeau, members of that body, and they have arrived at the conclusion, that the process of M. Brun Buisson is the best and most economical. It is as follows: take of the bruised root of valerian, two pounds; water, eight pounds; sulphuric acid, three ounces and one drachm; macerate for two days, and distil until the liquid no longer reddens litmus paper. The distilled fluid is then to be exposed to the air for a month, at the end of which time it is to be put into a matrass with half an ounce of recently precipitated, perfectly pure, hydrated oxide of zinc. This is allowed to digest for from eight to ten hours on a sand bath, heated to 176° Fahrenheit, and stirred occasionally. The warm liquid is filtered, and, after being evaporated to three-fourths of its volume, the residue is poured into porcelain capsules, and exposed to the heat of a stove. The product of this evaporation is half an ounce of valerianate of zinc in pearly crystals, in a state of perfect combination. The rationale of this process agrees with the opinion of M. Soubeiran, that the essential oil of valerian is converted into valerianic acid by oxidation, and that the acid has no previous existence in valerian root. The valerianate of quina may be prepared by a similar process, substituting pure quina for the hydrated oxide of zinc.

*The Valerianate of Zinc* appears to be a most valuable addition to the materia medica, combining the properties of an antispasmodic and a tonic, and, consequently, being peculiarly adapted for the treatment of neuralgic affections. Devay, who has employed it very extensively, states, that he has found it most useful in the treatment of facial neuralgia and of vertigo. After a fair trial of the remedy in many cases, we can confirm his observations, as also the fact noticed by him that this new chemical combination proves much more beneficial than the oil of valerian and oxide of zinc prescribed together. The high price of the salt unfortunately prevents clinical observations from being made in charitable institutions as to its effects. Valerianate of zinc is very readily decomposed, most acids setting free the valerianic acid, and combining with oxide of zinc. It also undergoes partial decomposition if exposed to the air, or even if kept in badly-stoppered bottles, when it emits a strong valerian odour—the perfect salt having but a very feeble odour, and being not completely soluble in water. The best characteristics of its purity are, its being in brilliant, pearly, tabular crystals of a snowy whiteness; its neutrality to litmus paper; its complete solubility in water, and its possessing but a very feeble odour of valerian. The dose of it is from three-fourths of a grain to one grain twice or three times a day; it may be prescribed in the form of pill made with a little mucilage, or conserve of red roses; or in solution in orange-flowers. The compounder must bear in mind that the crystals of valerianate of zinc do not dissolve readily in cold water, floating on the surface in consequence of their lightness; they should, therefore, be first incorporated with a few drops of water in a mortar.

*The Valerianate of Quina* may be prescribed in the same doses as the valerianate of zinc; it is more permanent in composition than that salt, and is equally soluble in water. It appears superior as an antiperiodic to disulphate of quina, in consequence of its neurosthenic properties; it is also given in much smaller doses, from six to ten grains being ordinarily sufficient to administer in the interval between the fits.—*Dublin Quarterly Journal.*

